# ‘Smart’ BLE wearables for digital contact tracing in care homes during the COVID-19 pandemic—a process evaluation of the CONTACT feasibility study

**DOI:** 10.1186/s43058-023-00533-0

**Published:** 2023-12-04

**Authors:** Carl A. Thompson, Amrit Daffu-O’Reilly, Thomas Willis, Adam Gordon, Catherine Noakes, Kishwer Khaliq, Amanda Farrin, Andrew Kemp, Tom Hall, Chris Bojke, Karen Spilsbury

**Affiliations:** 1 School of Healthcare, University of Leeds, Leeds, LS2 9JT UK; 2 Trials Research, Leeds Institute of Clinical, University of Leeds, Leeds, LS2 9JT UK; 3 Division of Medical Sciences and Graduate Entry Medicine, University of Nottingham, Derby, DE22 3NE UK; 4 School of Civil Engineering, University of Leeds, Leeds, LS2 9JT UK; 5 School of Electronics and Electrical Engineering, University of Leeds, Leeds, LS2 9JT UK; 6 South Tyneside Council, South Shields, NE33 2RL UK; 7 Academic Unit of Health Economics, School of Medicine, University of Leeds, Leeds, LS2 9JT UK

**Keywords:** Long-term care, Care homes, BLE Wearables, Digital, Contact tracing, COVID-19, Process evaluation, Normalisation Process Theory

## Abstract

**Background:**

Rapid and mass transmission of the SARS-CoV-2 virus amongst vulnerable people led to devastating effects from COVID-19 in care homes. The CONTACT intervention introduced Bluetooth Low Energy ‘smart’ wearable devices (BLE wearables) as a basis for automated contact tracing in, and feedback on infection risks and patterns to, care homes to try and improve infection prevention and control (IPC). We planned a cluster randomised controlled trial (RCT) of CONTACT. To be feasible, homes had to adopt CONTACT’s technology and new ways of working. This paper reports on the process evaluation conducted alongside CONTACT’s feasibility study and explains why it lacked the feasibility and acceptability for a definitive RCT.

**Methods:**

This mixed method process evaluation used Normalisation Process Theory (NPT) qualitative (interviews, field notes, study case report forms and documents, and observation) and quantitative (survey instruments, counts of activity) data to plan, implement, and analyse the mechanisms, effects, and contextual factors that shaped the feasibility and acceptability of the CONTACT intervention.

**Results:**

Thirteen themes within four core NPT constructs explained CONTACT’s lack of feasibility. Coherence: the home’s varied in the scale and extent of commitment and understanding of the technology and study procedures. Leadership credibility was important but compromised by competing priorities. Management and direct care staff saw CONTACT differently. Work to promote (cognitive participation) and enact (collective action) CONTACT was burdensome and failed to be prioritised over competing COVID-19-related demands on time and scarce human and cognitive resources. Ultimately, staff appraisal of the value of CONTACT-generated information and study procedures (reflexivity) was that any utility for IPC was insufficient to outweigh the perceived burden and complexity involved.

**Conclusions:**

Despite implementation failure, dismissing BLE wearables’ potential for contact tracing is premature. In non-pandemic conditions, with more time, better co-design and integration of theory-driven implementation strategies tailored to care homes’ unique contexts, researchers could enhance normalisation in readiness for future pandemic challenges.

**Trial registration:**

ISRCTN registration: 11,204,126 registered 17/02/2021.

**Supplementary Information:**

The online version contains supplementary material available at 10.1186/s43058-023-00533-0.

Contributions to the literature • This paper evaluates, for the first time, the implementation processes behind digital wearables for contact tracing in care homes, named CONTACT, using Normalisation Process Theory (NPT) to aid development and interpretation of the intervention ahead of a planned cluster RCT.

• Unlike previous research set in simulated or healthcare environments, care homes present unique challenges such as unavoidable close contact, high rates of people living with cognitive impairment and/or dementia, few tracing alternatives, and varied research support. This under-studied setting is the focus of our process evaluation, which elucidates why CONTACT was not feasible using a theoretical framework.

• The findings underscore the importance of timely, co-designed interventions and demonstrate how rapid implementation without considering external factors and the setting’s context can compromise even well-theorised and evidence-based interventions.

## Background

COVID-19 severely impacted long-term residential care, including nursing and residential care homes. At least 17% of the 274,063 deaths of residents and 14% of the 9175 deaths of care personnel in England and Wales between March 2020 and February 2022 were COVID-19 related [1, 2]. SARS CoV-2’s infectiveness, close proximity when delivering care, and resident frailty amplified risks [3].

Care homes often applied infection prevention and control (IPC) measures indiscriminately, with uncertain benefits and risks of harm for residents and staff [4–7]. Contact tracing reduces infections by identifying and managing individuals after contact with infected people. Its effectiveness depends on speed and comprehensiveness [8, 9]. Contact tracing may help control other diseases common in care homes, such as influenza and norovirus [10]. Despite COVID-19 vaccines, the need for non-pharmaceutical IPC, including contact tracing, remains [11].

Contact tracing in care homes is challenging. Traditional approaches rely on recall, analysing documents, or using smartphones. These are impractical in care homes: ~ 70% of residents have memory problems [12], documentation may be unreliable [13], and smartphone coverage is low [14].

**Fig. 1:**
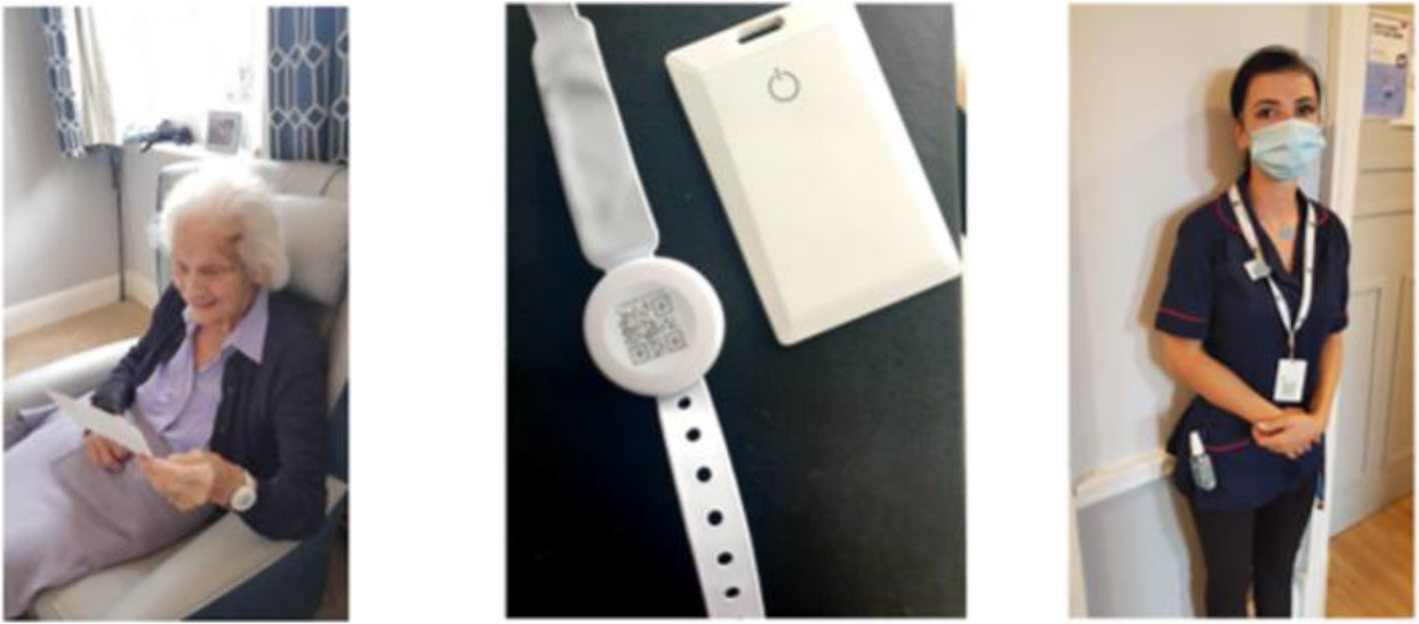
BLE wearable forms in a care home

BLE (*Bl*uetooth *E*nabled) wearable systems use low frequency wide area networks/LoRaWAN and the Internet of Things (IoT) to collect and transmit contact data: who, when, duration, proximity, and location. Wearables can be worn in fobs or as cards on lanyards (see Fig. 1). They have potential for analysing proximity networks [15], informing models of infection spread in long-term care [14], and as a swift, automated, scalable contact tracing method.

### The CONTACT study and intervention

CONTACT’s intervention was a BLE wearable system combined with location markers (static BLE devices) to map contacts. Devices and location markers had a unique identifier for de-anonymisation by homes. To examine wearable form effects and trade-offs (e.g. size vs. battery life), two homes (3 and 4) received fob-type devices and two (1 and 2) card-type devices.

A contact—two or more devices within 2 m for 15 min or more [16]—was transmitted to a Long-Range Wide Area Network (LoRaWAN) gateway via a ‘wave’ device [17] to our commercial partner’s (MicroShare®) [18] network. Anonymised data was forwarded to our Clinical Trials Research Unit, who analysed and summarised contacts, trends, and infection risks as the basis for feedback to homes.

Feedback was (i) structured monthly reports (Additional file 1) and (ii) ‘triggered’ reports generated when the research team was informed of a COVID-19 positive staff member or resident (Additional file 2). Feedback was individualised and aggregated: who had contact with whom, when, where, the duration of contact, average contacts, COVID-19 risk, and where contacts occurred. Reports were based on principles of effective feedback [19] and co-developed with managers and champions from the homes. Structured reports took two working days to generate and deliver to homes. Triggered reports were generated and delivered to homes within two hours of notification. They evolved based on staff feedback: for example, inserting key messages and simplifying visual representations of trends. Reports were emailed to homes and a researcher contacted homes to answer any queries 3 days later. Interactions between home and researchers were documented.

### Theoretical basis

BLE wearables generate data useful to care home decision makers for better informed IPC decision making [20, 21], reduced infection transmission, and use of potentially unnecessary restrictions. CONTACT was a novel technology for homes. It required new skills and new work. Accordingly, Normalisation Process Theory (NPT) [22, 23] and Rogers’ Diffusion of Innovation Theory [24] informed our intervention planning and evaluation. NPT emphasises four key constructs: coherence (sense-making), cognitive participation (engagement), collective action (work done), and reflexive monitoring (appraisal) [25].

A feasibility study of the intervention and planned RCT procedures was undertaken to inform decisions about the commitment of public funds. CONTACT did not proceed to a full RCT. Our analysis of the technical performance of BLE wearables (published elsewhere) showed adequacy when well implemented [26]. This paper evaluates CONTACT’s intervention and study implementation processes.

### Aim

The aim was to examine the processes and mechanisms influencing CONTACT’s intervention implementation and planned RCT study procedures.

## Methods

The process evaluation was conducted in four care homes in North and West Yorkshire, UK. All homes were for-profit, different sizes and registration status (Table 1). We first interacted with these homes in August 2021, with the formal feasibility study undertaken from November 2021 to February 2022, as COVID-19 restrictions were changing: homes were adapting to the OMICRON variant (November 2021), the imposition of ‘Plan B’ (face masks, vaccine passports, work from home from December 2021), and withdrawal of ‘Plan B’ (January 2022) [27].

**Table 1:** Care home characteristics

**Home**	**Type**^b^	**Ownership** [28]	**Maximum resident capacity**	**Number of staff**	**Number of residents**	**Number of residents with dementia (%)**	**Device type**^**a**^** issued**
Home 1	Residential	For-profit independent	30	25	26	6 (23%)	Card
Home 2	Residential	For-profit independent	15	21	15	2 (13%)	Card
Home 3	Nursing	For-profit independent	28	37	23	5 (22%)	Fob
Home 4	Dual residential and nursing	For-profit Non- Private Equity chain	102	120	87	25 (29%)	Fob

^a^Homes were allocated BLE wearables in card or fob-type forms (see Fig. 1)

^b^Residential care homes are safe environments for support with personal care: dressing, washing, and activities and opportunities for socialising. Nursing homes provide registered nursing care for those with higher levels of care need: post hospital discharge or with long-term care needs arising from conditions such as dementias. Nursing homes have a qualified nurse on site round-the-clock, supported by care assistants to provide higher levels of care

Ours was a mixed-methods process evaluation design exploring implementation processes, potential mechanisms of impact (of the intervention and the implementation), and contextual factors [29]. We employed a triangulation [30] design (qual|quant) [31] to ‘obtain different but complementary data on the same topic’ [30]. Qualitative data collection (interviews, field observations) approaches were broader in scope and able to accommodate unanticipated lines of enquiry. Quantitative approaches ([NoMAD] survey scores, counts [of devices worn] and summaries of key implementation processes [e.g. training attendance]) were more focused and structured. Neither qual or quant approaches were privileged analytically. Rather, they addressed similar phenomenon from different perspectives for a richer picture; for example, self-reported behavioural compliance with study procedures vs. the observed reality of compliance. The NoMAD survey [32] was used as a structured approach to explore change at two time points in (theoretically) important variables. It was used comparatively and as a heuristic to ensure we systematically and consistently captured NPT constructs.

Participants were selected based on their roles in implementing CONTACT and its study procedures: home managers, study champions care staff, and residents. Study champions were individuals nominated by homes to lead CONTACT study tasks, advocate for the study, and be a point-of-contact between home and research team; they received no payments. Homes usually had one champion but home 4 had two: an HR lead and an activities coordinator. Homes received a nominal fee per participant for taking part [33]. Staff received no direct incentives.

We collected qualitative (interviews and field observation) and quantitative (survey scores, counts, and summaries of key implementation processes) data.

- Care home interviews: Semi-structured interviews explored intervention/study understanding, engagement, enactment, and comprehension. Interview schedules were designed around NPT constructs. Thirty-eight interviews (see Table 2) were conducted. Interviews with managers, deputies, and study champions were conducted online due to COVID-19 restrictions and their personal preference. Care staff and residents were interviewed in care homes as COVID-19 restrictions allowed. Interviews varied from 25 min to an hour and were conducted, recorded, and transcribed verbatim by two researchers. Interviews were in weeks 6–8 of the intervention. Follow-up interviews with managers took place after the intervention ended.
- Field notes: Researchers maintained records of care home interactions: meetings, training sessions, phone calls.
- Field observation [34]: Two pragmatic field observation exercises were conducted in two care homes to compare our records of BLE wearable uptake with observed data and note study protocol deviations.
- Quantitative study data: the NoMaD survey tool, a validated measure of NPT [32] (sub)domains, was used at the beginning and end of the intervention with each home manager. Counts of training sessions attended, log completion rates.

**Table 2:** Participant interviews by home

**Home**	**Manager**	**Nurse**	**Carer/assistant**	**Well-being**^**b**^	**Admin/other**	**Resident**	**Total**
1	1^a^		4		1	4	10
2	1^a^		2			3	6
3	1^a^		2		1	4	8
4	1	1	2	2^a^	2 + 1^a^	5	14

^a^Denotes a study champion role

^b^‘ Well-being’ includes informal (hairdressing appointments) and formal (weekly exercise classes) activities coordination in the home

### Data analysis

Qualitative data were organised using Microsoft Excel and analysed using a framework analytic approach and NPT-informed coding matrices derived from NPT guidance NPT for developing and evaluating complex interventions [35–37]. Phase 1 had five stages, first familiarising ourselves with transcripts, field observation notes, and survey and count data (stage 1). A thematic framework was generated using deductive and inductive coding of a subset of data (see Table 3), ensuring emergent themes were included (stage 2). We applied this framework to other data (stage 3) and summarised each source’s findings (stage 4). Finally, we synthesised and interpretated data, focusing on patterns and connections (stage 5). Initial analysis was by ADO with themes refined by AS and other team members.

**Table 3:** NPT constructs, themes, and illustrative data/analysis

**NPT construct**	**Theme | subconstruct(s)**	**Illustrative data/analysis**
Coherence | sense making	Variable buy-in | communal specification	*“Staff and residents had a lack of understanding. My understanding wasn’t there, and I can’t expect someone to understand something that I don’t understand myself” (home 1, study champion)*
	Legitimacy and credibility | individual specification	*“No investment from staff, it was not engrained within in the care home enough. As much as we could tell them to wear them, there are more than 100 people. I think it was up to the leads to encourage staff to wear the device, and that approach wasn’t there. The staff didn’t really remember or care to do it.*” (home 4, study champion)
	Across-role engagement | individual and communal specification	Managers and senior staff demonstrated understanding and engagement, others had minimal understanding and engagement
	Carer engagement | individual specification	*“I wear my device at all times, but I know others take theirs off …” (home 2, care assistant)*
Cognitive participation | work to promote CONTACT engagement	Identifying and appointing the *right* key staff | initiation | enrolment	In three smaller homes, managers took on champion roles as there were no staff judged to have the requisite skills
	Finding and engaging gatekeepers for whole home engagement initiation | enrolment | activation |	Against advice, one home appointed multiple study champions. In three smaller homes, managers assumed study champion roles and struggled to enact work required. Staff were gatekeepers (of variable quality) for recruiting and retaining resident participation
	Enacting study tasks | legitimation |	Variable staff commitment meant key study tasks (CRFs, device logs, battery records) were variably completed
	Diverse motivations | legitimation	Motives for participation were not always COVID-19 related
	Acceptability and wearability | legitimation	Some staff removed devices when undertaking key personal care (assisting with feeding or personal hygiene). Some resident devices in suboptimal locations masking contacts (handbags, cupboards and drawers). Managerial estimates of compliance (~ 80% wear) did not match observed reality (7% in one 15-min observation period of 41 people in a communal area)
Collective action | individual’s CONTACT enactment work	Balancing workload against available resources interactional workability	*“…difficult to prepare for such a big workload when one doesn’t know what’s coming. Don’t know until you do it. Wouldn’t have put us off, but we would have been better prepared”* (home 4, manager)
	Training and support from a distance | relational integration | skill set workability (training)	Remote and virtual training led to attendance of between 33 and 100% (mean 65%)
	Credibility of CONTACT data | relational integration | relational integration (disruption)	*“I wasn’t confident with some of the data on the scheduled report because the locations were showing people were having contacts and congregating in the corridors, and I know for sure that they don’t meet there. So that was lacking in the accuracy, a lot of the contacts in my home happen in rooms, like day rooms and dining rooms”* (home 3, manager) *“The scheduled reports seem to replicate what was happening, it made sense as it showed staff were supposed to be where they should be. That give me the confidence it was picking up the people it should. It then translated into confidence that it would be a useful tool to monitor where the infections were and how they would be transferred”* (home 1, manager)
Reflexive monitoring | appraising CONTACT	Negative feedback learning loops and balance | communal and individual appraisal	*“The triggered report covered mostly what we knew already. I did analyse the scheduled report which identified which residents are most at risk. But if you find out which individuals are most at risk, what can you really do with that information? We can make people isolate but then you lose staff. The staff do a lateral flow test before work every morning, that’s the protection we already have, without losing too many staff”* (home 4, study champion)

Phase 2 involved aligning themes with NPT’s four core constructs and associated sub-constructs (for example, *Coherence*: differentiation | communal specification | Individual Specification | Internalisation) to validate findings and check for inconsistencies. The process was flexible enough to accommodate new themes, for example, limited ‘pull’ from care homes for ‘pushed’ [38] CONTACT-generated information [39]. Two researchers (ADO, AS) checked coding choices; disagreements were resolved through discussion with the study lead (CT).

Quantitative data (counts, proportions, summary measures of central tendency and variation) were generated using R and SPSS (version 21) statistical software packages and treated non-inferentially.

We used between-method triangulation of methods (for example, comparing expected device counts from records and interviews to observed counts from field notes and observation) to provide a more comprehensive understanding of implementation processes [40]. Our team included two experts by experience—family carers—who helped formulate evaluation plans and interpret results.

## Results

Tables 1 and 2 detail key home and participant characteristics.

Thirteen themes within four core NPT constructs were generated.

### Coherence: CONTACT sense making | differentiation | communal specification | individual specification internalisation

#### Variable buy-in

Commitment to CONTACT varied, and comparisons with alternatives such as ‘Test and Trace’ were often made. For some, the (potential) advantages of CONTACT were clear:

“The CONTACT trial is just there and monitors more quietly in the background. Whereas “test and trace” you have to go through that torture, months after getting notifications.” (home 4, care assistant).

For others, less so:

“I would say they (care staff) were indifferent to the study, not positive. They don’t see the positive effect that the devices could have.” (home 4, study champion 1).

Home 3’s manager and champion were strongly committed: sharing study information with staff, encouraging participation, responding promptly to issues, and regularly contacting the research team. Home 4 manager’s commitment was less evident, and whilst their champion was engaged, staff shortages limited her involvement.

Care homes 1 and 2’s managers were also study champions—ensuring some degree of commitment. But home 1’s manager left abruptly, causing significant disruption, poor staff understanding and engagement, and a study champion with a gap in their ‘coherence’:

“Staff and residents had a lack of understanding. My understanding wasn’t there, and I can’t expect someone to understand something that I don’t understand myself” (home 1, study champion).

#### Legitimacy and credibility

The authority and credibility of those who introduced and endorsed CONTACT in the homes significantly impacted uptake and engagement.

“No investment from staff, it was not engrained in the care home enough. As much as we could tell them to wear them, there are more than 100 people. I think it was up to the leads to encourage staff to wear the device, and that approach wasn’t there. The staff didn’t really remember or care to do it.” (home 4, study champion).

Home 3’s study champion was well respected, and the manager reinforced CONTACT’s legitimacy by communicating its importance to staff. In contrast, the absence of a strong advocate in home 1 weakened the study’s legitimacy.

#### Engagement across roles

Engagement with the study varied across roles. Senior staff and managers demonstrated understanding and engagement, mainly as they were involved with study tasks and communications. Staff least likely to receive devices (night and agency staff) often showed minimal knowledge of CONTACT.

“I think the reach with the agency staff that we got at the start were okay, but the devices that we got later. I don’t think any of the agency staff actually participated. I think because of the boxes with the signing out sheets would have been left in the nursing rooms, so I think it is the difficult of the senior in charge thinking about the agency devices with everything else.” (home 4, study champion 1).

Residents’ recall of CONTACT’s purpose was very limited. Many accepted BLE wearables without hesitation. But most could not recall why they were wearing the device. For some, the device became ‘part of their routine’, whilst others were unaware of its existence. Despite being able to demonstrate understanding and capacity to consent to wearing devices during recruitment, some residents misunderstood device purposes, for example, thinking they were fall alarms.

“The staff understood what we were doing and were happy to wear the devices, but I think the residents didn’t fully understand why they were wearing them and found them annoying to wear.” (home 2, manager).

#### Care staff engagement

Care staff engagement with CONTACT was generally high, but not always:

“I wear my device at all times, but I know others take theirs off …” (home 2, care assistant).

“On our floor the staff fully understood it. Some of us decided to opt in while others opted out.” (home 4, care assistant).

Some staff admitted to ‘forgetting’ their devices (their emphasis) or deliberately taking and leaving them at home.

Care staff engagement was weakened by staff perceptions of data. Staff spoke of being perceived as, ‘not doing their jobs properly’ (champion, home 4) because CONTACT appeared to reveal limited staff movement in the home. Others erroneously believed devices could track them outside the home. We combatted this misinterpretation through messaging and the champions and managers. Concerns lessened as staff understanding of CONTACT’s purpose spread and improved.

### Cognitive participation: work promoting CONTACT engagement initiation | legitimation | enrolment | activation

#### Identifying and appointing key staff

Identifying study champions was an essential part of CONTACT’s implementation and evaluation plans. In one home, the manager selected *two* study champions to manage research coordination. The manager felt a *third* study champion would have increased study feasibility further, as CONTACT meant deferring champions’ other work tasks.

“There needs to be a contract tracing team or ambassador, they can do admissions or consents, the only thing they do is CONTACT. Yes, either it is one person’s job or it’s a whole home approach were everyone is in on it. Everyone knows to wear a device, and the leads know to make sure people are wearing their device. I think however you do need both.” (home 4, study champion 2).

Conversely, and despite researchers’ warnings of potential pitfalls when managers also assumed champion roles, the three smaller homes’ managers/deputies opted to also be study champions, compelled by a perceived dearth of staff with the right skills and availability.

“I think that I was the only person that could have driven it forward to be honest … The resources and capacity as far as the typing in of the forms and at the beginning the logic and process skills that you have to have to fill out your master log; check out your data to send it to you; put your data back in, record it in an ordered manner, that’s not your typical social care worker. They don’t do anything like that or record anything. They just live in the moment. You need very on the ball manager to actually handle it all.” (Home 3, Manager).

Champions, drawing on factors such as permanent employment contracts, leadership roles, and established staff rapport, believed their roles were well positioned to advocate for and manage CONTACT.

“I would say it’s entirely my baby and they’ve allowed it because nobody wants to take any more on anyway … I think they (staff) roll their eyes and think “what is [manager] up to now?” Not in a bad way, we like to try new stuff.” (Home 3, Manager and champion).

Home 1’s manager-champion left abruptly, compromising adequate and efficient training provision and research activity understanding and spread. To keep the home in the study, the research team had to adapt and simplify research procedures. In the past, the home was supported by in situ researchers, working in the home. This was seen facilitative, and the absence of it a key deficit of CONTACT’s approach.

### Engaging the ‘whole home’ and gatekeepers

Champions, in conjunction with the research team, used various strategies to foster engagement: a ‘Change of Practice’ notification, electronic mail introductions, social media group networks, as well as tangible (pens) and electronic promotional materials, and staff meetings.

Only one home utilised our ‘verbal and opt-out’ ethical approval (home 3). This strategy saved time but relied heavily on trust between manager and care teams. Study champions in other homes approached interested staff individually. All the champions and managers suggested increased researcher presence would have facilitated better engagement. Recognising that COVID-19 restrictions made this unrealistic.

Obtaining written consent from residents was assigned to care staff rather than researchers. For residents lacking capacity, champions enacted processes to contact nominated consultees. This was labour-intensive, which required multiple phone calls and providing written information. Consultee unresponsiveness created further study delays.

To improve intervention understanding, the research team developed a staff guide on using, maintaining, and cleaning CONTACT’s devices for champions to disseminate. A brief version came with each device to foster engagement and familiarity. Problems with study maintenance (battery changes) and care (reports of dirty devices, possible infection risk) were common in homes—especially home 4, the largest but least engaged.

### Enacting study tasks

Training helped champions with key tasks such as recruitment and screening. But homes often required additional remote and/or virtual support, walkthroughs, or reminders from the research team. Champions were proactive and confident in seeking guidance from the research team.

We needed staff to wear the device, ensure its visibility (to help accuracy and reinforce adoption), take it home (to avoid extraneous/false contact data), and assist residents with their devices. Managers expressed confidence (probably erroneously) that most consented participants wore the devices:

“… So compliance not so good in the beginning, better now than what it was. I think 85% of the time people have had it on when they needed to have it on.” (home 4, manager).

A good understanding of the study did not produce consistency in enacting key study tasks such as completing weekly logs, notifying the research team of loss or breakage, and reporting positive COVID-19 cases.

“I would say the staff did understand what was required, I just don’t think they were able to do all the things required consistently.” (home 4, study champion 1).

Non-champion participants often struggled to consistently adhere to study protocols due to competing work tasks and routines. The frequent turnover of staff and the employment of agency staff further reduced consistency.

### Diverse motivations

Staff motivations to participate in CONTACT ranged from mild interest in the study, managerial influence, indifference, to a desire to combat COVID-19 and enhance resident safety.

“I wouldn’t say staff have been interested. There hasn’t been a lot for them to do. They just wear the device. They just knew what we were doing and they were going to do it that’s it. They were participating for the care home manager not for the interest in the project.” (home 2, manager).

Yes, my role is to protect our residents so anything that can have a positive impact that’s our job to look after them. (home 3, care assistant).

Amongst those who could recall, residents’ reasons for participation included the desire to contribute to COVID-19 work, a belief in potential future benefits, or just wanting to participate with no clear rationale.

I thought it might do some good. (home 1, resident).

Staff and resident have mostly got on with it. There is one lady that won’t come out of her room without it, it’s part of her routine now. We spoke to the residents and she was really up for it. She likes to help as much as she can, she believed it would help with COVID. (home 2, manager).

The extended timeframe between consent and start of the intervention (mean time from consent to issuing resident devices was 41 days (SD = 23.87)) was compounded by residents’ memory problems.

### Acceptability and ‘wearability’

Wearing the device when undertaking routine care work was challenging for staff and residents. Staff often removed externally worn devices to prevent accidental contact with residents, contamination with food, or obstructing daily tasks (for example, providing personal care or food preparation).

Most staff wore devices consistently and adhered to protocols, but not always. Even when staff understood study requirements (coherence), they admitted to wearing them inside pockets or leaving them in work lockers. Device management and daily study operations were resource-intensive work and not always successful. In the largest home, power cables were lost, and a wave scanner went missing for a week. In the smaller (#1) home, residents unplugged infrastructure components (waves).

Many residents removed devices, storing them in drawers, tissue boxes, or handbags, reducing data accuracy [26]. Staff sometimes attached devices to residents’ belongings, such as walking frames, and were confused about managing devices ‘found’ in the home. Some residents found devices uncomfortable. Residents with dementia particularly struggled with consistent device usage. Something some staff took as a default:

Half of the residents can’t wear them as they have really bad dementia and they would just grab them off. (home 2, care assistant).

I don’t think it would work for every care home, for example we had difficulty with residents who had dementia. Similar homes may struggle, as it is a danger to put the device on dementia residents or they don’t know why they are wearing it. It may work in some homes but not others. I think also if the devices were designed a bit differently there wouldn’t be as many issues with wearing them. (home 2, study champion).

One manager (home 1) noted residents did not consistently wear their devices and staff and that encouraging residents diminished over time. Conversely, staff were *more* consistent:

The residents also forgot to wear them often. The longer we were going the less care was taken to see if the device was with the resident as they were attached to different things. It was hard to keep the residents continuously wearing them but staff wore it throughout their shift. (home 1, manager).

Observation in two homes revealed only a small fraction of staff and residents wore devices correctly. Despite managers’ accounts of high device usage (~ 85% or more) and fidelity, many were not worn visibly on wrists or lanyards. One 15-min observation session of 41 people (23 staff and 18 residents) showed only two staff and one resident wearing devices. Despite inconsistencies, staff often indicated wearables were part of ‘daily routine’, only noticeable when they were obtrusive. Interestingly, we noted occasions where champions and managers were not wearing devices. Reasons given by staff for withdrawing residents from the study included residents not wanting to wear one or feeling distressed or confused by them.

### Collective action—CONTACT enactment work by individuals interactional workability | relational integration and disruption | skill set workability assignment and training | contextual integration managerial support | resources

#### Workload vs. available resources in a changing context

All homes described CONTACT’s workload as substantial, demanding, and burdensome, particularly participant screening log completions, obtaining consent, and registering participants. These tasks were complicated on paper by staff and hindered by paper resident data and rudimentary digital infrastructure. Whilst most screening was completed within the required period, the largest home needed more time: more participants meant more work. Some study champions extended working hours to complete study-related tasks:

I find I have to shuffle things around to make it work. When things were heavier, I would usually finish at 5, but during the screening and consent time I had to stay late at night to contact the families. It was hard it fit it in, into an already hard day (home 4, study champion 1).

Securing resident consent or assent from consultees was time-consuming. Homes devised strategies, such as intercepting next of kin on planned visits. Such ‘work arounds’ were not scalable:

At the beginning it was tricky to get the consents of all the residents. It was a large workload and I underestimated that. I could go into one bedroom to see the resident and be in there for 30 min and still not obtain the consent. For relatives, I had a spreadsheet of how many times I have contacted them and what they said. That was quite stressful as we had a deadline to get all the consents. I would wait until after 5 pm when I finished my work to call. Some of them never replied so it meant I had to change my tactic by waiting at reception if I knew the relative was visiting the care home. (home 4, study champion 2).

After initial screening, consent, registration, and device distribution, staff focused on device management and data collection. This involved regular tasks like submitting weekly reports on new participants, withdrawals, deaths, device management, and battery replacements. They also had to keep a daily log of COVID-19 cases, report cases, and attend a weekly call with the research team to review reports, monitor progress, and address issues. Despite ‘burdensome’, ‘time-consuming’, and inconsistencies reports from homes, they mostly completed tasks on time. Weekly support calls from the research team were highlighted by champions and managers as ‘essential’. As time progressed and knowledge increased, some homes (#3 and #4) were able to submit weekly data independently.

Having committed to the study, most felt capable of ‘handling the work’, but only for the duration of the feasibility study. Despite consent explanations, the full extent of study involvement was only clear after starting:

“ … difficult to prepare for such a big workload when one doesn’t know what’s coming. Don’t know until you do it. Wouldn’t have put us off, but we would have been better prepared” (home 4, manager).

The pandemic context shifted. We recruited homes pre-vaccination but delivered CONTACT post-vaccination. One manager detailed how they ‘made room’ for CONTACT due to the urgency of the research:

“Because of the nature of why I took it on and being in the middle of COVID, I didn’t have the capacity. But the importance to me of doing it made me make the capacity. I still would argue I don’t have the capacity, I know I’m speaking to you today, so I’ve made sure all my forms are done, so I do need to have this weekly checkpoint, otherwise I would easily drift. It’s the most involved study I’ve done where I’m involved doing it and collating and understanding it. But the importance of the study outweighs what I would do” (home 2, manager).

Fob battery replacement was an unforeseen and laborious task. Collecting devices, changing batteries, returning the devices, and logging changes were time consuming for staff. Staff in home 3 managed to change batteries themselves, but delays in home 4 meant a researcher going in to change 34 batteries for the staff over 2 days.

Field notes from the largest home confirmed the complexities of managing study recruitment. Balancing different tasks and apprehension about the daunting task of resident recruitment resulted in delayed and rushed ‘last-minute’ recruitment. Despite advice from researchers that this task should be undertaken first due to its time-consuming nature, the champion did not respond to emails and calls and a (self-declared) ‘sense of panic’ resulted.

Study procedures (and CONTACT’s perceived complexity) were complicated further by research governance. Each device had a unique number that was cross-referenced against each home’s ‘master log’ to identify the wearer from anonymised reports. Communication involved secure file transfer of non-identifiable data. The secure databases used for registering participants and reporting COVID-19 cases experienced technical difficulties—introducing more delays and effectively ‘negative feedback’ for homes that had spent time reporting cases and expected a rapid response.

#### Training and support for homes at a distance

COVID-19 restrictions prevented almost all in-person visits or training in the homes. An introductory session and three shorter online ‘micro training’ sessions covering study activities were offered. Whilst all participants found the training comprehensive, some found it too intense. Table 4 reveals the adequate but variable uptake of training of key staff.

**Table 4:** CONTACT training uptake

**Home**	**Invited**	**Attended**	**%**
#1	9	3	33.33%
#2	4	4	100.00%
#3	7	5	71.43%
#4	14	10	71.43%
**Total**	**34**	**22**	**64.71%**

#### Credibility of CONTACT data

Confidence in CONTACT’s triggered and scheduled reports (Additional files 1 and 2) varied. Staff were sceptical regarding the accuracy of contact locations and the devices (individuals) involved. Staff recalled instances where contacts occurred in ‘unusual’ places or between people who would ‘not usually interact’. Doubts that led some staff to believe devices could record contacts through walls and ceilings, generating further scepticism:

“I wasn’t confident with some of the data on the scheduled report because the locations were showing people were having contacts and congregating in the corridors, and I know for sure that they don’t meet there. So that was lacking in the accuracy, a lot of the contacts in my home happen in rooms, like day rooms and dining rooms” (home 3, manager).

One home’s lack of confidence in CONTACT data hindered report sharing and led them to consider withdrawing:

“The data collected didn’t reflect what was happening in the home. The devices seemed too sensitive. I was therefore dubious of the data. I lost a bit of faith and questioned what I was doing. One of the reports showed two residents who have never had contact did have contact, but their rooms are directly above each other” (home 4, manager).

Conversely, when reports *confirmed* expected contact patterns, confidence in reports improved:

“Scheduled reports seem to replicate what was happening. It made sense, as it showed staff were supposed to be where they should be. That give me the confidence it was picking up the people it should. That translated into confidence that it would be a useful tool to monitor where the infections were and how they would be transferred” (home 1, manager).

“The triggered report was helpful as it confirmed what we suspected. One resident for example said that was positive, her neighbour goes into her room a lot and we see this in the report and a staff member that seen her on the day. We tested both individuals, and both were (COVID-19) positive” (home 4, manager).

Despite study reports providing insights into contact patterns, they did not impact on IPC protocols or behaviours. Staff felt current practices, aligned with national guidelines, were effective enough, despite the uncertainty associated with national and local IPC guidelines.

#### Reflexive monitoring—appraising CONTACT | reconfiguration

CONTACT reports were largely confined to home managers/champions. Consequently, staff were unfamiliar with the information and its potential benefits, resulting in lower engagement and fewer opportunities for learning. The largest home was wary about acting on reports because highlighting staff behaviours was problematic:

“We can’t do much with the triggered reports… We can see that a person is in the smoking area for so long, but we can’t approach them to say anything because that would decrease their trust and they wouldn’t wear the devices. That would then spread to other people as they think they’re being tracked” (home 4, study champion).

Staff saw daily testing as adequate protection from infection *without* threatening staffing levels or relationships, whereas CONTACT’s information was harder to operationalise and risked making scarce (human) resources scarcer.

“I did analyse the scheduled report which identified which residents are most at risk. But if you find out which individuals are most at risk, what can you really do with that information? We can make people isolate but then you lose staff. The staff do a lateral flow test before work every morning. That’s the protection we already have, without losing too many staff” (home 4, study champion).

Home 3’s manager welcomed the study’s triggered report when understanding the infection source of two COVID-19 positive staff. Since no other cases were identified, the infections were likely acquired outside the home. In home 4, the report supported the view that an isolated resident’s lateral flow test result was a false positive—later confirmed by polymerase chain reaction (PCR) tests. Managers thus recognised the *potential* utility of the data for establishing isolation zones, escalating testing, and preventing home-wide shutdowns. But crucially, outside a research study context:

“…I can see in the future how it could work, preventing us having to close because we’ve got two cases out of 80, we can easily isolate pockets of people if we needed to and staff as well. So, I can see if we didn’t have the national guidelines in place, where it would give me research-based information to make risk assessment decisions. If this wasn’t a trial and we had this info because this was the system we were using, I would feel comfortable saying, “hang on a minute, this is showing, this is showing and this is what we can do about it” as an assessment to present to anybody. In the guidelines, it does say that registered managers are accountable for decisions. Outside of a trial, it would have given me the confidence to say this is what the infection is doing, and we can safely isolate and carry on doing what we are doing with the other residents so residents don’t suffer from lack of visitors” (home 3, manager).

Non-wearable CONTACT technology (location markers and wave routers) became familiar and routine. Wearing devices consistently did not. One manager’s reflection suggested that normalisation was a possibility—albeit with dedicated support:

“I think if it is part of infection prevention strategy then you have got someone running it then it would become part of that strategy and part of the way we work. I think if it was a dedicated person’s role, and they had a team of assistants [in the home’s communities] then it would work… I think if we were to carry on from the point that we are at now, it would be more recognised and more part of the day-to-day stuff. That’s how people have started to think about it now. If we went on for another year at the level we are at now, it would be common practise for residents and staff. More time to become normalised, that’s the right word” (home 4, manager).

Table 5 details NOMAD scores from home managers at feasibility start and end. Limited patterns include:

- Familiarity: At the start, all care homes felt somewhat to completely familiar with using devices. Care homes CH1 and CH2, however, did not provide full data. CH3 showed an increase in familiarity over time, whilst CH2 showed a decrease. CH4 remained consistently high in their comfort level.
- CONTACT as regular work: In CH3 and CH4, there was an increasing trend in considering CONTACT as a normal part of work by the end-point. Looking to *future* normalisation, CH3’s belief diminished.
- CONTACT’s (potential) value: Most homes saw the potential value of CONTACT, supporting its ongoing use and being open to working in new ways for CONTACT.
- Resources and training: Whilst care homes generally believed sufficient training was provided, they varied regarding the availability of resources. CH3, for example, showed a decline in its perception of available resources over time.
- Integration and disruption: Responses varied regarding the ease of integrating CONTACT into existing work: CH2 and CH3 found it more challenging to integrate by the end-point. Furthermore, some homes felt CONTACT disrupted working relationships (CH3).
- Management and feedback: Almost all homes believed management adequately supported CONTACT and that feedback *could* be used for future improvements.
- Awareness and valuation of effects: CH3 held off on expressing a view based on lack of relevance at study start, but agreement in the value of CONTACT’s effects, potential for formative feedback, and work modification was stronger by study end. Overall belief in valuing CONTACT’s effects varied across the homes.

**Table 5:** Home manager’s NOMAD assessments

**NoMaD item**		**Care home (CH) and time point (start/end)**						
	**CH1 (start)**^**a**^	**CH1 (end)**	**CH2 (start)**	**CH2 (end)**	**CH3 (start)**	**CH3 (end)**	**CH4 (start)**	**CH4 (end)**
When you use devices, how familiar does it feel^b^	10	-	8	5	6	10	8	10
CONTACT is currently a normal part of your work^b^	5	-	5	5	6	7	8	10
CONTACT will become a normal part of your work^b^	10	-	2	-	9	7	10	10
Differs from usual ways of working^c^	7	-	3	2	2	2	3	2
Staff have a shared understanding^c^	2	-	2	2	2	3	3	3
I understand how CONTACT affects the nature of my work^c^	3	-	2	3	2	2	2	2
I can see the potential value^c^	1	-	2	3	1	1	1	1
There are key people who drive CONTACT forward and get others involved^c^	1	-	3	2	2	2	1	1
I believe that participating in CONTACT is a legitimate part of my role^c^	4	-	2	2	2	2	1	1
I am open to working with colleagues in new ways to use CONTACT^c^	1	-	2	2	2	2	1	1
I will continue to support CONTACT^c^	1	-	7	3	2	2	1	1
I can easily integrate CONTACT into my existing work^c^	1	-	2	4	2	4	1	1
CONTACT disrupts working relationships^c^	4	-	4	3	4	4	5	5
I have confidence in other people’s ability to use CONTACT^c^	1	-	2	4	2	4	4	3
Work is assigned to those with skills appropriate to CONTACT^c^	4	-	3	3	2	2	1	1
Sufficient training is provided to enable staff to implement CONTACT^c^	2	-	1	2	2	2	2	1
Sufficient resources are available to support CONTACT^c^	2	-	1	2	3	4	2	1
Management adequately support CONTACT^c^	1	-	1	3	2	2	2	1
I am aware of reports about the effects of CONTACT^c^	2	-	1	3	7	2	2	1
The staff agree CONTACT is worthwhile^c^	2	-	3	4	7	3	2	2
I value the effects of CONTACT has had on my work^c^	2	-	3	4	7	2	2	2
Feedback about CONTACT can be used to improve it in the future^c^	2	-	2	2	2	2	1	1
I can easily modify how I work with CONTACT^c^	2	-	3	3	3	3	1	1

^a^CH1 did not provide end data

^b^Rated from 0 (unfamiliar) to 10 (completely familiar)

^c^1 = strongly agree, 2 = agree, 3 = neutral, 4 = disagree, 5 = strongly disagree, 6 = not relevant for my role, 7 = not relevant at this stage

In summary, NOMAD revealed generally positive views on the implementation and potential value of CONTACT. But this general picture masks variation, especially concerning resource availability, integration challenges, and perceived disruption.

## Discussion

CONTACT’s intervention and study procedures were not implemented successfully. Whilst technology infrastructure installation, care home recruitment, and care home support were positive, sustaining commitment, a sense of legitimacy in the technology, data, and information provided was not. Our findings mirror similar evaluations of technology intended to reduce pandemic COVID-19 infections in social care. For example, Ullah et al. [41] saw 88% *less* use of a symptom tracker in care homes over time and no impact on spread or infection rates [41].

To the best of our knowledge, CONTACT is the first evaluation of BLE wearables for contact tracing in care homes during the pandemic. The process evaluation and associated theoretical lens of NPT helped explain the individual, team, and home levels and external or ‘outer-contextual’ factors [42] that hindered implementation.

The context in which CONTACT happened was crucial [43, 44]. COVID-19 restrictions, staffing pressures, and changing national and local IPC guidance, whilst anticipated, necessitated continual pragmatic adaptation of plans and execution. Whilst these effects may have undermined the foundational components required for normalisation, the pandemic also compromised potential solutions. Facilitation and facilitation skills have been suggested as ways of working *with* context during a study [43]. But even with ‘generic’ facilitation expertise present in our research team, the pandemic context and adjustments to relational working within the complex social systems of homes meant this key element of implementation—and thus CONTACT’s impact—was sub-optimal [45]. Whilst the pre-vaccination pandemic context provided urgency to homes’ IPC efforts, they were also short-staffed and faced with an ever-changing panoply of new COVID-19 mitigation and management tasks. CONTACT was simply another component in a ‘burden bundle’ (manager, home 4) that had to be managed to normalise CONTACT. Our findings provided insight into each of NPT’s four core components: coherence, cognitive participation, collective action, and reflexive monitoring [25].

The care home communities’ diverse range of motivations for participation, varied understanding of CONTACT’s ‘value proposition’, the technology itself, and how homes’ systems should adapt given their readiness for adoption suggest c*oherence*, or sense-making, was limited. Such failures are not limited to NPT-based explanations (cf. Greenhalgh’s NASS model or Roger’s Innovation Adoption characteristics) [24, 42, 46].

Homes’ *cognitive participation*, or work to foster engagement, varied across homes and participants. Study procedures and remote reliance on homes meant delays between participant consent and individual activation may have weakened initial buy-in from homes. But once in situ and implementation commenced, motivation continued to dwindle. Others have highlighted the effects of time and evaluation stages on potential for deviation from interventional procedures (programme drift) or diminishing effects as the distance from design stages, through evaluation, to real-world use, increases (voltage drop) [47]. As the need for greater cognitive participation *given* the (sub-optimal) implementation approaches employed increased, we saw less compliance. Contact tracing needs sufficient and consistent population coverage to be successful [14]. We were in effect setting a trial up for lower effectiveness. Our results suggest that employing alternatives to linear conceptualisations of technology use as a pipeline of ‘development—efficacy—effectiveness—real world use’ may yield better compliance generally and effective contact tracing specifically. Pre-engagement and ‘in situ’ iterative, engagement, and formative learning that fosters trust between homes and researchers may be more useful [48].

The *collective action* work required for implementation depended on CONTACT aligning with existing workflows [45]. However, the burdensome nature of the study tasks for homes and the perception amongst some staff that BLE wearables hindered already normalised work meant suboptimal device use. Given our remote implementation plan, the pandemic and planned RCT contexts CONTACT was unlikely to be realised. The process evaluation revealed that managers’ reported compliance and observed wearable and study procedural compliance differed. Such gaps, revealed by research methods, are not new [49]. More naturalistic and ethnographic research methods may have produced a stronger foundation for understanding how collective action could have been nurtured to support implementation [23, 49, 50]. The pandemic context and associated restrictions prevented us using these methods.

Though some managers’ reflexive monitoring led them to express the potential benefits of CONTACT, the real-time impact on infection prevention control (IPC) protocols was minimal. Arguably, reflecting the tension between balancing (innovative) interventions against established practices where the ‘relative advantage’ [24] of the intervention are (a) not immediate, (b) not always clear, and (c) occur in a complex system where the outcomes of the intervention may themselves be uncertain. Not everyone who came into contact with those testing positive for COVID-19 was themselves infected, quickly, and IPC was known to be imperfect [51]. Views arising from reflexive monitoring could be considered an artefact of our testing of rigorous (but burdensome) RCT procedures: ‘the trial killed the intervention’ [48]. Murray and colleagues suggest a more optimistic role that NPT-based evaluations such as CONTACT highlight that the intervention and associated implementation plans did not justify the costs likely to be incurred by rushing to a definitive cluster trial; thus, CONTACT was (entirely justifiably) a ‘trial killer’ [36].

### Other theories of technology implementation failure

Our NPT-focused analysis describes and explains CONTACT’s implementation failure. But other models and frameworks would also likely yield a similar diagnosis and prognosis for the technology in its post-vaccination, RCT-testing, context. Additional and unanticipated work arising from issues such as fob battery replacement and checking data accuracy would have limited likely adoption according to the Technology Acceptance Model [52]. Similarly, applying Socio-Technical Systems thinking would have emphasised the importance of a harmonious fit between CONTACT (as technological ‘solution’) and the socio-cultural environment of the homes [53, 54]. In our case, the challenge of integrating CONTACT into daily routines, coupled with socio-cultural barriers such as staff concerns over ‘being monitored’, hampered adoption.

### Limitations

CONTACT was devised, developed, and commissioned quickly: from inception to commencement in 4 months. Whilst demonstrating that partnerships between academia, tech and care industries can be developed quickly, such rapid commissioning in a pandemic context necessitated compromise in the design and implementation co-production. Co-creating technologies can increase the depth and scale of adoption and spread of technologies in care homes [55]. Co-creation that happened post-deployment did so in the context of adaptation, refinement, and workarounds for limitations and expressed burden. Our study encapsulates (and validates) Peryer and colleague’s [56] assertion that without adequate co-development, the ‘trial risks killing the intervention’ [56, 57]. Staff ‘shock’ at CONTACT’s workload could have been mitigated if we had used relatively simple questions to aid home selection—acknowledging care homes unique context [58]. Such risk-appraisal may mitigate the effects of homes’ premature enthusiasm for research in a future pandemic without increasing the risks of non-delivery from excessive demands on scarce resources.

Despite our multi method approach, we drew heavily on reported behaviours. We were prone to the same biases and limitations that led to the need for technology-enabled contact tracing in the first place: residents were limited in their ability to recall rationale or past events, and staff may provide biased responses. Social desirability bias cannot be ruled out. Staff knew CONTACT was associated with IPC and so positive behaviours may have been exaggerated and deviance downplayed. Emphasising views that align with preconceived beliefs and expectations (confirmation bias) and a reluctance to report negative (or indeed, overly positive) views because of fear of retribution or negative consequences was also possible.

Finally, our evaluation period of 2 months—due in part to the success of the UK’s vaccination programme—was a too limited window for the technology to be become ‘normalised’ and embedded into everyday work in each home.

## Conclusion

CONTACT did not become part of everyday work in care homes in a pandemic. We did not develop and sustain sufficient coherence in our messaging and education around the technology; roles were enacted and adapted based on partial or mis-information and tended toward mechanistic study requirements rather than fostering and sustaining in situ usefulness of the technology; the resources and mechanisms we offered to help the homes overcome barriers to normalisation were insufficient for overcoming the perceived (for example, resident inconvenience) and actual (regular battery changes and associated study requirements) limitations of the technology; and, finally, the relatively short time scale of 2 months of full intervention in a post-vaccination context meant learning reinforced negative perceptions that the technology lacked utility for IPC.

However, concluding BLE wearables have no promise or cannot become part of normal work in care homes would be premature. With caveats, we provided social network data to UK SAGE—Scientific Advisory Group for Emergencies—Social Care subgroup and enabled the UK PROTECT National Core Study on COVID-19 to examine air quality and ventilation in care homes [59].

COVID-19 meant the research team had to resort to implementation methods we knew were suboptimal. In a non-pandemic context, with sufficient time and planning, a theory-based plan for optimising for the challenges, barriers, and levers in care homes could result in greater normalisation of the technology. The technical performance of the wearable technology used was adequate for useful contact tracing, if BLE wearable systems were adapted and co-developed *with* homes as part of planned implementation to encourage the requisite shared understanding of how data from wearables will be used and the actions that could be taken as a result; BLE wearables may yet play a part in the response to pandemics that we know will emerge in the future.

### Supplementary Information

**Additional file 1.** Structured monthly reports.

**Additional file 2.** Reactive ‘triggered’ report example.

## Data Availability

Interview schedules are available on request from the corresponding author.

## Abbreviations

BLE: Bluetooth enabled
RCT: Randomised controlled trial
RSSI: Received signal strength indicator
NPT: Normalisation Process Theory
NoMaD: Normalisation Measure Development questionnaire
NHS: National Health Services
NIHR: National Institute for Health and Social Care Research
CONTACT: CONtact TrAcing in Care homes using digital Technology
IPC: Infection prevention and control
SARS-CoV-2: Severe acute respiratory syndrome coronavirus 2
COVID-19: Coronavirus disease
IoT: Internet of Things
GPS: Global positioning system
LoRaWAN: Long-Range Wide Area Network
ENRICH: Enabling Research in Care Homes
NICHE-Leeds: Nurturing Innovation in Care Home Excellence in Leeds
CQC: Care Quality Commission
CRF: Case report form(s)
