# Supplementary material for: ‘Smart’ BLE wearables for digital contact tracing in care homes during the COVID-19 pandemic—a process evaluation of the CONTACT feasibility study
Source: Implement Sci Commun. 2023 Dec 4;4:155. doi: 10.1186/s43058-023-00533-0 (PMC10694939; doi:10.1186/s43058-023-00533-0)
Supplement: Supplementary file 1 — Additional file 1. Structured monthly reports. [file 43058_2023_533_MOESM1_ESM.pdf]

What does your CONTACT data show?

REPORT 3 - 18/02/2022

Here are contacts* between residents, staff and visitors in your home since the previous report. This report highlights who, where and when contacts are happening and how contacts are changing over time. It also provides information on any infections occurring in your home.

CONTACT team researchers will call you in a few days to talk through the information. They can help you understand what, if any, action you might like to consider.

**CONTACT is two people within 2 metres for 15 minutes or more*

Residents with the most contacts

15 (83%) of residents had contact with **another resident**

17 (94%) of residents had contact with **a staff member**

32 (82%) of staff had a contact with **a staff member**

Device ID	Resident	Staff	Visitor
AC233FA8BD68	18	26	0
AC233FA8BD23	15	22	0
AC233FA8BD1A	10	26	0
AC233FA8BD2B	13	22	0
AC233FA8BD0C	12	20	0
AC233FA8BCFB	14	16	0
AC233FA8BD17	10	20	0
AC233FA8BCFA	8	18	0
AC233FA8BD1C	9	14	0
AC233FA8BD1F	12	10	0

Most contacts — Least contacts

Changes in contacts over time

The bars and lines on these graphs show different information. The bars show the total number of unique device pairings each week. Example: Device A records contact with Device B three times, Device A records contact with Device C five times, Device B records contact with Device D six times - the unique number of pairings is three.

The dots/lines in the graph show the average number of contacts that each device has had in one week. Example: like before, Device A records contact with Device B three times, Device A records contact with Device C five times, Device B records contact with Device D six times. The average is calculated by dividing the total number of contacts each device has had - 28 - by the number of devices - 4. So, the average number of contacts is 7

Resident - Resident

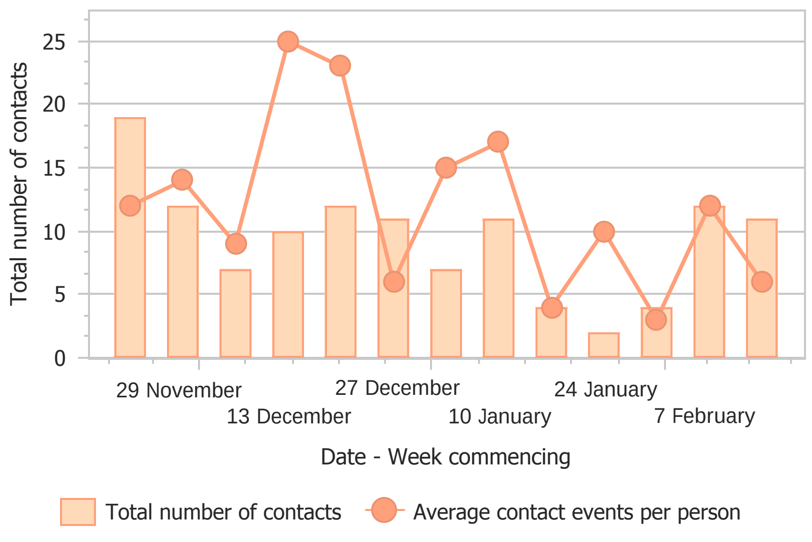

Key Message

The total number of pairs of resident-resident contacts each week has remained relatively stable with a dip towards the end of January.

The average number of contacts per resident has fluctuated over time showing no particular pattern.

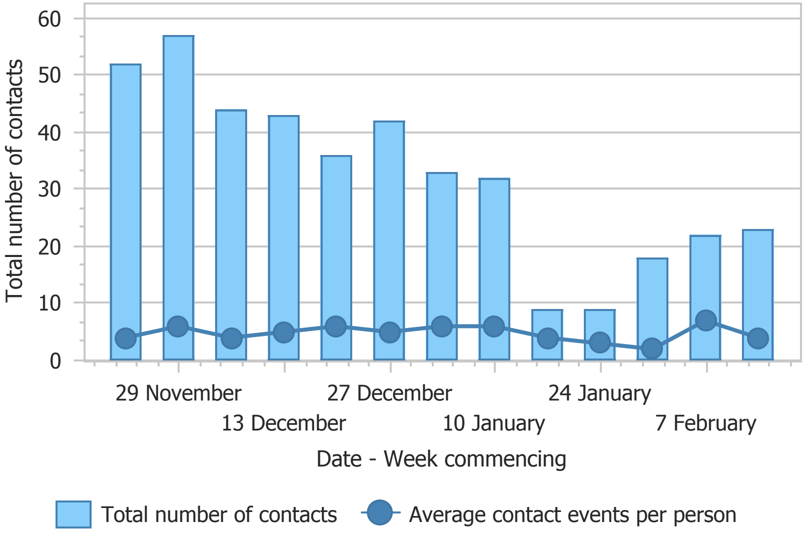

Resident - Staff

Key Message

The total number of pairs of resident-staff having contact each week showed a steady decline until end January when it started to increase again.

The average number of times residents have had contacts with staff has however remained relatively stable.

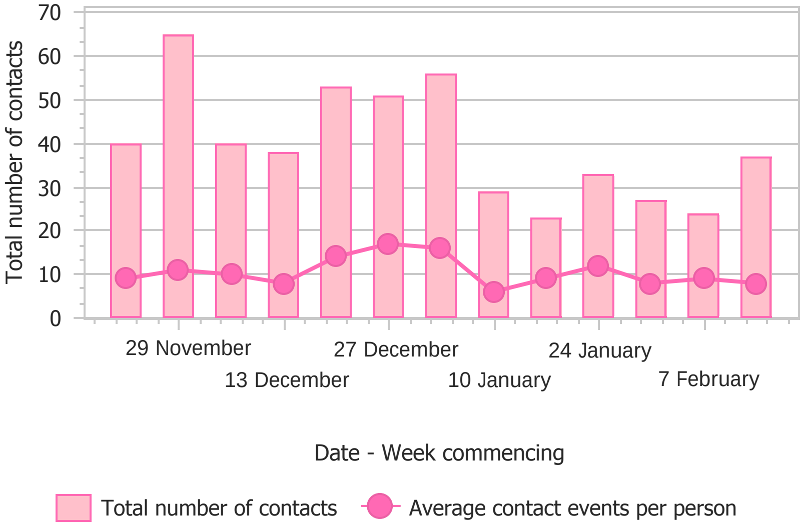

Staff - Staff

Key Message

The total number of pairs of staff-staff having contact with each other has remained relatively stable since the beginning of January. The average number of times in a week that staff are having contact with each other has also remained reatively stable at around 10 contacts each per week.

Where are the most contacts taking place?

Resident - Resident

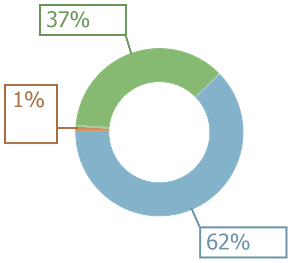

Resident - Staff

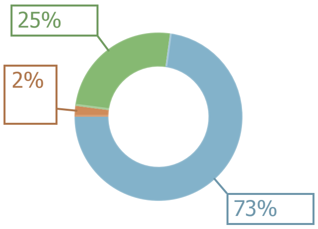

Staff - Staff

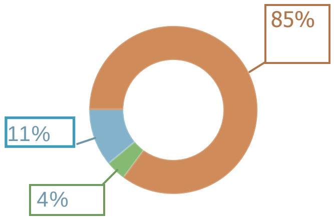

Corridor   Reception   Lounge   Dinning Area   Office   Staff Room

Who are at most risk of infection?

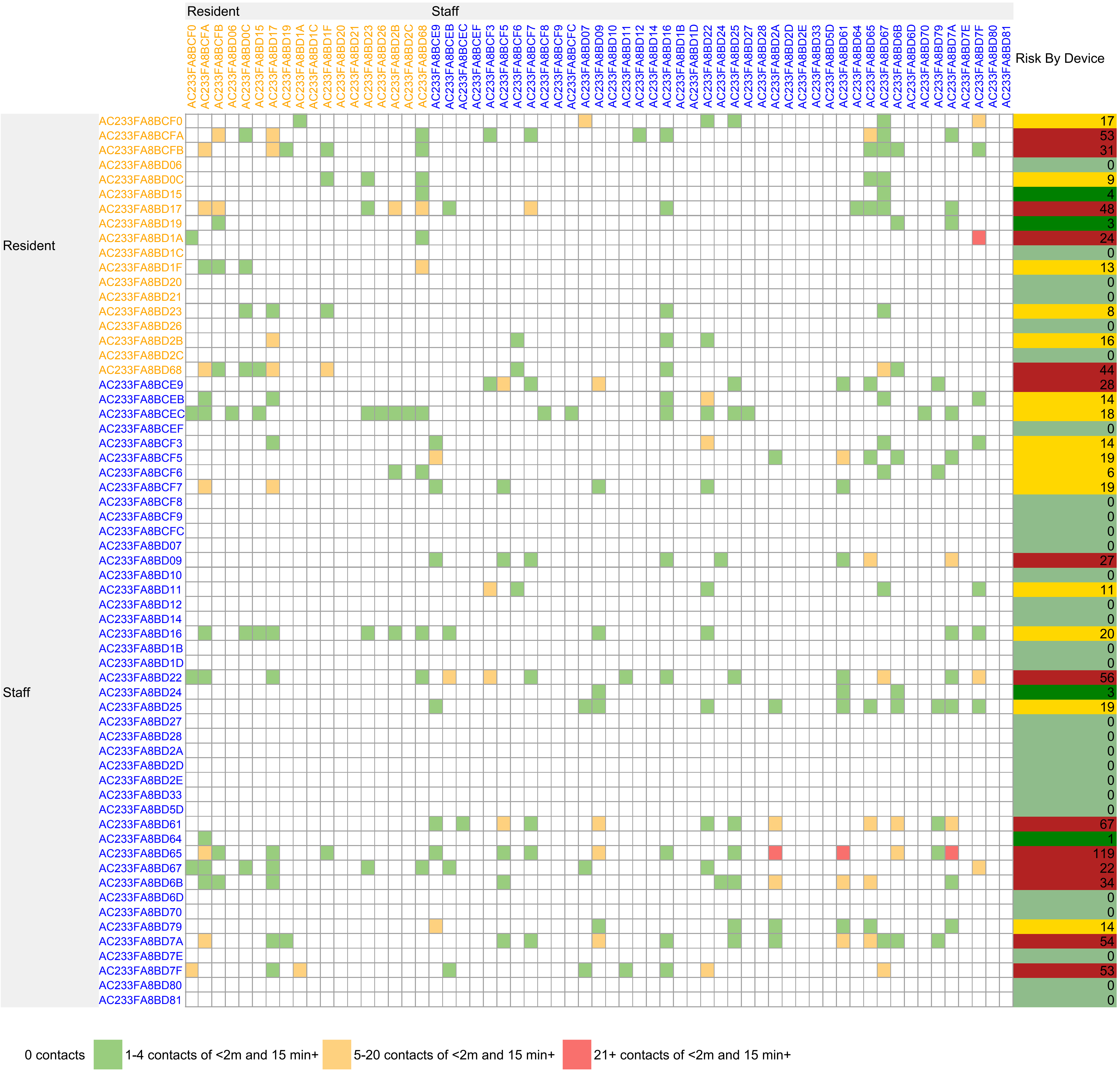

Number of days without an infection

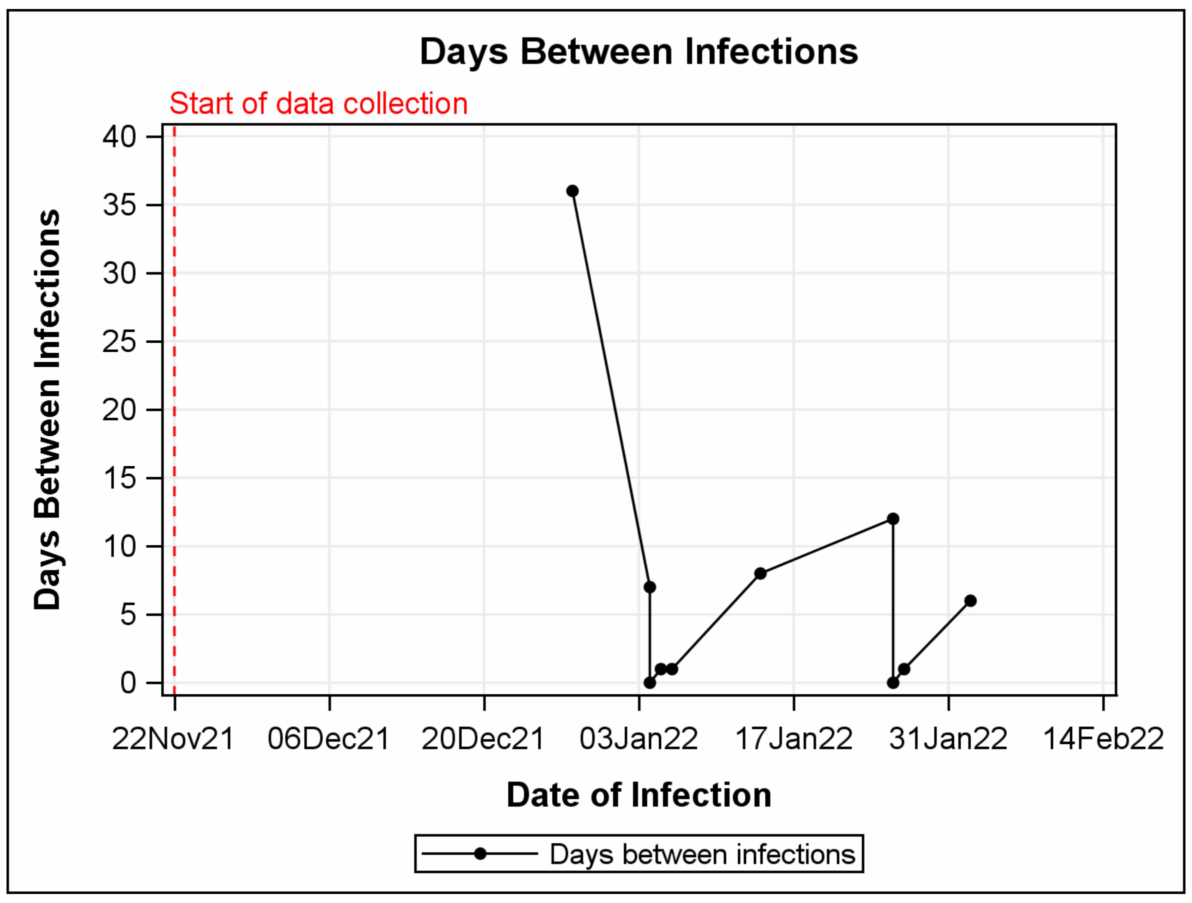

Key message

Since the start of data collection you had over 35 days before your first reported infection. However, it has been less than 14 days between subsequent infections. The goal is to increase the number of days without an infection.
