# Supplementary material for: ‘Smart’ BLE wearables for digital contact tracing in care homes during the COVID-19 pandemic—a process evaluation of the CONTACT feasibility study
Source: Implement Sci Commun. 2023 Dec 4;4:155. doi: 10.1186/s43058-023-00533-0 (PMC10694939; doi:10.1186/s43058-023-00533-0)
Supplement: Supplementary file 2 — Additional file 2. Reactive ‘triggered’ report example. [file 43058_2023_533_MOESM2_ESM.pdf]

Appendix B: Reactive "triggered" report example

CONTACT
Contact tracing in care homes using digital technology

University of Leeds

Triggered Report

For the period 01/02/2022 – 03/02/2022

The CONTACT tracing technology currently in your care home transmits data relating to the number of contacts made in the home – a contact is when 2 or more devices come into contact with each other for 15 minutes or more at a distance of 2 metres or less.

This report provides you with a breakdown of those contacts.

The first page/s provide you with a general 'at a glance' quick summary of the headlines. It tells you who has come into contact with who, how many times and where. This type of information can help you to identify 'hot' areas where a lot of people might be coming into close contact with each other. The device IDs are already matched up to residents, staff and visitors. You can use your Master Device Log to find out more specific information about which device belongs to which person.

The 'detailed report' provides a more detailed breakdown of the 'at a glance' summary. This tells you about who came into contact with who, how long the contact was, what time the contact started, when it ended, and where the contact took place.

A quick reminder on how to interpret the report:

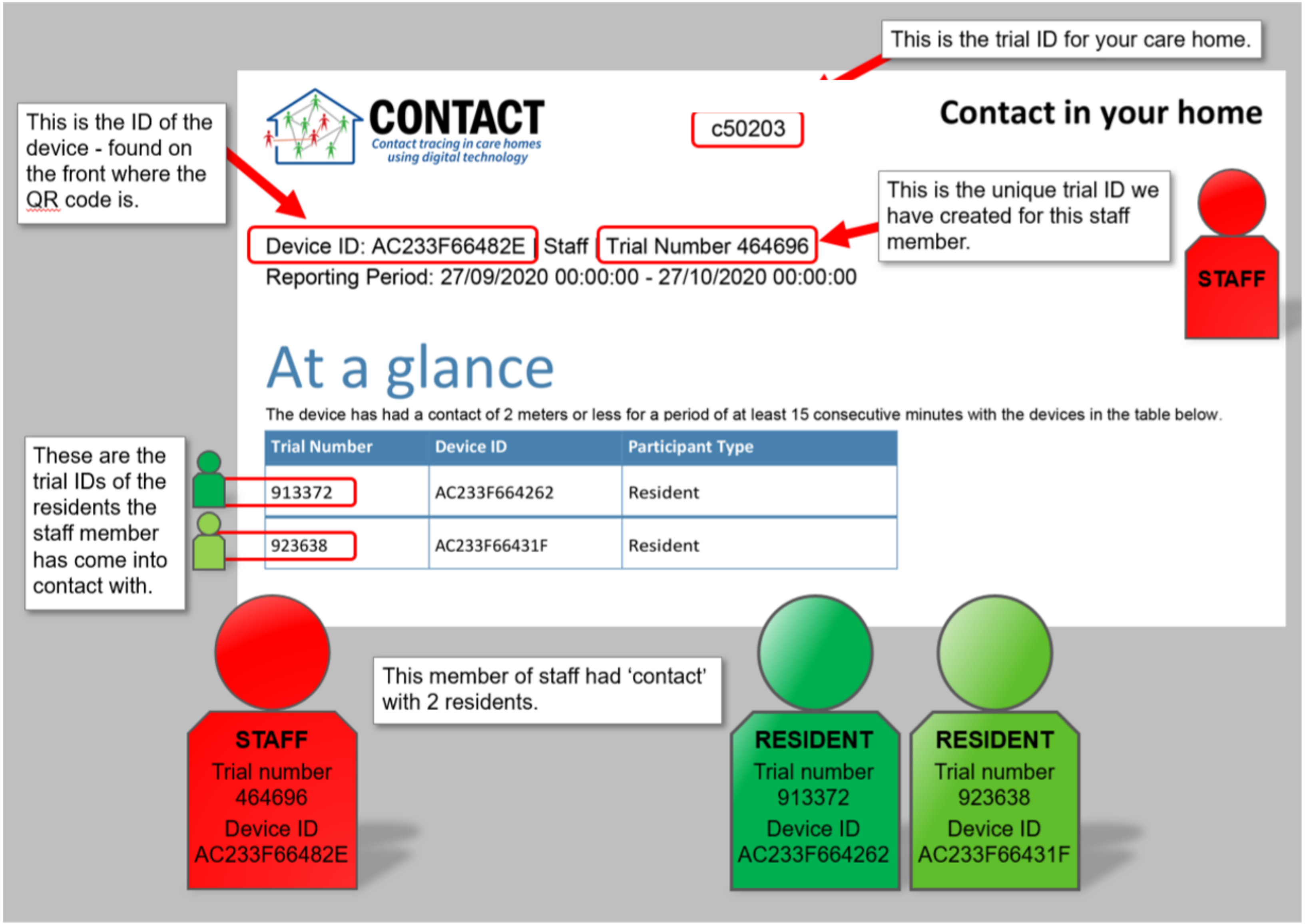

Trial Number	Device ID	Participant Type
913372	AC233F664262	Resident
923638	AC233F66431F	Resident

This view has the contacts that were summarised before, now separated out into each individual contact.

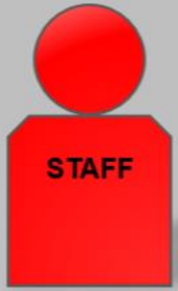

Full Contact Details

The table below shows all contacts the device has had. Please refer to local visitor logs for any visitor devices shown in this report.

Device ID: AC233F66482E | Staff | Trial Number 464696

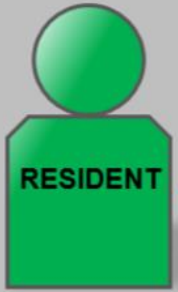

Device ID: AC233F664262 | Resident | Trial Number 913372

Duration (Minutes)	Start Time	End Time	Room
11	08/10/2020 10:03	08/10/2020 10:14	1st Floor - Canteen behind TV
11	08/10/2020 10:03	08/10/2020 10:14	1st Floor - Canteen behind TV
11	13/10/2020 09:07	13/10/2020 09:18	2nd Floor - Kitchen behind TV
11	13/10/2020 09:07	13/10/2020 09:18	2nd Floor - Kitchen behind TV

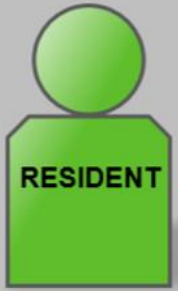

Device ID: AC233F66431F | Resident | Trial Number 923638

Duration (Minutes)	Start Time	End Time	Room
13	10/10/2020 11:03	10/10/2020 11:16	1st Floor - Canteen behind TV
13	10/10/2020 11:03	10/10/2020 11:16	1st Floor - Canteen behind TV
